# A simple three-input DNA-based system works as a full-subtractor

**DOI:** 10.1038/srep10686

**Published:** 2015-06-22

**Authors:** Hung-Yin Lin, Jian-Zhou Chen, Hao-Yi Li, Chia-Ning Yang

**Affiliations:** 1 Department of Chemical and Materials Engineering, National University of Kaohsiung, Kaohsiung, Taiwan; 2 Institute of Clinical Medicine, Medical College, National Cheng Kung University, Tainan, Taiwan; 3 Department of Life Sciences, National University of Kaohsiung, Kaohsiung, Taiwan

## Abstract

Over the past decade, DNA has demonstrated remarkable potential in fabrication of molecular logic and arithmetic systems. In this work, a simple DNA-based system mimicking a full-subtractor that handles three inputs including one minuend and two subtrahends for eight input/output conditions is successfully designed. The whole system is established by one gate molecule and three input sequences, all made of single-stranded DNA sequences.

As interest in nanotechnology research has grown, nanoscale devices, which can be designed using either a top-down or a bottom-up approach, have been widely studied^1^,^2^,^3^. In 1994, Adleman introduced a DNA-based biocomputing system for solving famous mathematic traveling salesman problems^4^. This led to additional explorations and experiments demonstrating that DNA sequences can serve as elementary computing devices such as half-adders^5^,^6^,^7^,^8^, half-subtractors^9^, full-adders^10^, and full-subtractors^11^. Related DNA-based computing experiments have involved smart molecular systems such as majority voting logic circuits^12^,^13^,^14^,^15^, keypad locks^16^, and multilevel logic circuits^17^,^18^,^19^.

In addition to providing these basic computing capabilities, effectively designed DNA molecules show promise for application in other functions such as molecularly targeted cancer therapy^20^,^21^,^22^. In designing nanoscale devices, many scientists prefer using DNA molecules over typical synthetic polymers because DNA molecules offer a wealth of favorable conformational characteristics that typical polymers lack. For example, DNA molecules can have single-stranded sticky ends or can form duplexes, and a DNA molecule may possess a hairpin loop, G-quadruplex, or crossover structures. DNAzyme molecules or aptamer–substrate complexes are also options for biomolecular designers^23^.

DNA-based computing devices are typically designed to mimic Boolean logic operations (such as AND, OR, XOR, NOR, NAND, XNOR and INHIBIT) that manage one or more logical inputs to produce a single logical output. Designers combine these types of logic gate to create logic circuits for arithmetic calculations. For example, the logic circuit of a half-subtractor requires two binary digits: I_A_ (the minuend) and I_B_ (the subtrahend) to perform subtraction and the involved outputs are a difference-bit (by using an XOR gate) and a borrow-bit (by using an INHIBIT gate). The value of the difference-bit is equal to the minuend subtracts the subtrahend, regardless of whether the value is positive or negative; if the minuend is less than the subtrahend, then the borrow-bit value equals 1. Considering four input states of (0,0), (0,1), (1,0), and (1,1) for 0 – 0, 0–1, 1–0, and 1–1, respectively, the INHIBIT operation outputs borrow-bit values of 0, 1, 0, and 0, respectively, and the XOR operation outputs difference-bit values of 0, 1, 1, and 0, respectively. For instance, when performing 0–1 = −1 represented by the input state (0,1), the outputs are difference-bit of 1 and borrow-bit of 1.

A full-subtractor is a combinational logic circuit that performs subtraction using inputs of three binary digits: one minuend and two subtrahends, and considers eight input states: (0,0,0), (0,1,0), (1,0,0), (1,1,0), (0,0,1), (0,1,1), (1,0,1), and (1,1,1). Similar to a half-subtractor, a full-subtractor outputs values for one difference-bit and one borrow-bit. For instance, the inputs (1,1,1) would be processed as 1-1-1 = −1, and would output a borrow-bit value of 1 and a difference-bit value of 1.

In this study, we extended our previously examined DNA-based half-subtractor systems^6^,^8^ by considering a third input strand, to form a full-subtractor that handles three inputs in eight input/output conditions. The full-subtractor proposed herein is designed to be relatively compact and composed of merely four DNA molecules. Three strands are as inputs: I_A_ (the minuend), I_B_ (the first subtrahend), and B_in_ (the second subtrahend) and one strand as a logic gate to recognize the input strand(s) and produce two sets of output signals obeying the truth tables of difference- and borrow-bits in a full-subtractor.

The design of the gate strand relies on a molecular beacon, which commonly serves as an optical DNA probe. A molecular beacon is a single-stranded DNA sequence with a loop and stem in a hairpin conformation, containing a quencher and a fluorophore, generally at the 3’ and 5’ ends, respectively. When a molecular beacon is in a hairpin conformation, its quencher and fluorophore remain in vicinity to each other, and the fluorescence signal is thus quenched. However, when a complementary DNA target is detected, the hairpin opens into a linear form and the fluorophore, now away from the quencher, releases an observable signal. When the gate strand is in hairpin form, the fluorescence signal is off and designated as 0; whereas in linear form, the fluorescence signal of the gate strand is on and designated as 1.

Herein, we used a gold surface to immobilize the molecular beacons and to replace the quenchers, because of the superior quenching capability of the gold surface for certain fluorophores^23^. Thus, the fluorophore is carried at its 3’ end, and its 5’ end is modified with disulfide for gold-surface immobilization. Fluorescein (green dye, λ_em_ = 520 nm) is linked at the 3’ end because its fluorescence is quenchable by the gold surface. All three inputs are also single-stranded DNA sequences. Based on most designs in related studies^5^,^6^,^7^,^8^,^9^,^10^,^11^, the presence and absence of an input strand at the gate molecule are assigned Boolean values of 1 and 0, respectively. Eight combinations of input strands may be applied to the logic gate, represented by the aforementioned eight input states.

Table 1 shows the DNA sequences used in the proposed full-subtractor. The gate molecule is a 29-nt DNA hairpin containing a 19-nt loop and a 5-nt stem, bearing a 5’ end-linked with disulfide for gold-surface immobilization and a 3’ end-linked with a fluorescein. The input strand I_A_ (the minuend) is 46 nt long carrying a BHQ3, a quencher to Cy5 (red dye, λ_em_ = 662 nm), at its 5’ end; the input strands I_B_ and B_in_ (the two subtrahends) are both 76 nt long, both carrying a Cy5 at their 3’ ends. The 27-nt sequence at the I_A_ strand 5’ end is complementary to the 27-nt sequence of the I_B_ and B_in_ 3’ ends, as shown in the boxed segments of the nucleotide sequences depicted in Table 1. The 19-nt sequence of the I_A_ strand 3’ end is complementary to the 19-nt sequence of the gate molecule 5’ end, as shown in the sequence segments in boldfaced font. The 19-nt sequences in the I_B_ and B_in_ strand middle regions are complementary to the 19-nt sequences of the 3’ end of the gate molecule, as shown in the wavy underlined segments. The 30-nt sequences at the I_B_ strand 5’ ends and the B_in_ strand 5’ ends are complementary to each other, as indicated by the dash-underlined segments.

Based on such sequence assignments, the gate molecule changes from hairpin to linear conformation upon recognizing input I_A_, I_B_, or B_in_; furthermore, the input strands show preference to form I_A_/I_B_, I_B_/B_in_, and I_A_/B_in_ duplexes when they are in proximity with the gate molecule. The fluorescein on the gate molecule is quenchable by the gold surface whereas the Cy5 on I_B_ and B_in_ strands is quenchable by the BHQ3 on an I_A_ strand. When the gate molecule is in hairpin form, the fluorescein emission is quenched by the nearby gold surface; however, when an input molecule is present, the gate molecule opens into linear form and the fluorescence from fluorescein is observable. When I_A_/I_B_ and I_A_/B_in_ strands are partially hybridized, Cy5 and BHQ3 are drawn into proximity, enabling the BHQ3 to quench the Cy5 signal. As I_B_/B_in_ hybridization occurs, fluorescence from Cy5 is released. The on/off patterns of released Cy5 and fluorescein are read as binary outputs for the borrow-bit and the difference-bit, respectively.

Figure 1(A) shows a schematic illustration of the proposed full-subtractor. The left half depicts the four input states in which the second subtrahend B_in_ = 0; thus, the operations of these four states resemble those of a half-subtractor system, which manages a minuend and only one subtrahend, represented by I_A_ and I_B_, respectively. In a (0,0,0) input state, no input is added and the fluorescein of the gate molecule remains near the gold surface; hence, no fluorescein signal is released. Both the borrow-bit and difference-bit are read as 0, as listed in the truth table in Fig. 1(B). In the (1,0,0) input state, input strand I_A_ is added, thus changing the gate molecule conformation from hairpin to linear. As the fluorescein at the 3’ end moves away from the gold surface, it releases a green fluorescence signal, corresponding to an output difference-bit status of 1 for 1–0–0 = 1. In the (0,1,0) input state, input strand I_B_ is added, thus disrupting the hairpin conformation of the gate molecule. In addition to the green fluorescence signal from the gate molecule, a red fluorescence signal from Cy5 on the I_B_ strand is also observable. Both the borrow-bit and difference-bit are read as 1, represented by 0–1–0 = −1. In the (1,1,0) input state, both the I_A_ and I_B_ strands are added. Because I_A_ and I_B_ are designed to have a higher probability of forming an I_A_/I_B_ duplex (as opposed to forming an I_A_/gate duplex or I_B_/gate duplex), the hairpin conformation of the gate molecule is not substantially disturbed, and the fluorescein signal is quenched by the gold surface. Meanwhile, the partial I_A_/I_B_ hybridization causes the BHQ3 of I_A_ to remain adjacent to the Cy5 of I_B_, thus quenching the Cy5 signal. The borrow-bit and difference-bit values are both read as 0, as indicated in the truth table in Fig. 1(B) for 1–1–0 = 0.

The right half of Fig. 1(A) shows the four conditions from the lower part of the truth table in Fig. 1(B), where the second subtrahend is B_in_ = 1. Including the B_in_ strand increases the complexity of the system, but its delicate interplay with the other strands enables the Cy5 and fluorescein on/off patterns to correspond to those listed in the Boolean truth table. In the (0,0,1) input state, the added B_in_ strand works in the manner of the added I_B_ strand, using the same nucleotide segment to hybridize part of the gate molecule and transform the gate molecule from hairpin to linear form. The (0,0,1) state switches on both the Cy5 and fluorescein signals, which correspond to 0–0–1 = −1, with values of borrow-bit = 1 and difference-bit = 1. In the (1,0,1) input state, the added I_A_ and B_in_ strands have a higher priority to hybridize each other, instead of interacting with the gate molecule (similar to the I_A_ and I_B_ interactions in the (1,1,0) input state). The I_A_/B_in_ partial hybridization draws the BHQ3 and Cy5 into close contact, which inhibits a Cy5 signal release. Both the Cy5 on the B_in_ strand and the fluorescein on the gate molecule are off (not released). This represents 1–0–1 = 0. In the (0,1,1) input state, the input strands I_B_ and B_in_ are included and they hybridize each other using their complementary 30-nt segments in the 5’ ends, which leaves the Cy5 signal at their 3’ ends on. The gate molecule remains in hairpin conformation and the fluorescein signal is quenched by the gold surface. This corresponds to 0–1–1 = -2, which is the “high borrow” condition, the only condition without an analogue in the truth table. In the (1,1,1) input state, the inclusion of I_A_, I_B_, and B_in_ generates a combination of the interactions associated with (1,1,0), (1,0,1), and (0,1,1), with I_A_/I_B_, I_B_/B_in_, I_A_/B_in_, I_A_/gate, I_B_/gate, and B_in_/gate duplex formations producing fluorescence signals released from both Cy5 and fluorescein. This corresponds to 1–1–1 = −1, with both the borrow- and difference-bit read as 1.

The truth table in Fig. 1(B) shows all possible input values, fluorescence on/off states (for Cy5 and fluorescein), and output values (borrow-bit and difference-bit). Figure 1(C) shows the measured fluorescence intensities, in arbitrary units (a.u.). The (0,1,1) input state produces a Cy5 fluorescence intensity that is nearly twice those in the (0,1,0), (0,0,1), and (1,1,1) input states because in (0,1,1) state there are two Cy5 sources (I_B_ and B_in_), instead of only one Cy5 source (either I_B_ or B_in_) in the other three input states. However, in the (1,1,1) state the Cy5 intensity is similar to those in the (0,1,0) and (0,0,1) states, but lower than that of (0,1,1) state, regardless the presence of I_B_ and B_in_ in (1,1,1). It is likely that half of I_B_ and B_in_ strands hybridize to I_A_ and their Cy5 fluorescence is therefore quenched. Meanwhile, the slightly low fluorescein intensity in the (1,1,1) state implies that most of the added input strands prefer to form I_A_/I_B_, I_A_/B_in_, and I_B_/B_in_ duplexes, although some strands remain available to interact with the gate molecule transforming the gate molecule into linear form, thus turning on the fluorescein signal. One possible way to increase the fluorescein intensity for the (1,1,1) state is to slightly enhance I_B_/B_in_ interactions over those of I_A_/I_B_ and I_A_/B_in_, so that adding the three input strands would result in more I_B_/B_in_ duplexes and fewer I_A_/I_B_ and I_A_/B_in_ duplexes, which would leave a greater portion of the I_A_ strands to interact with the gate molecule and release the fluorescein from the gold surface.

## Conclusion

This study presents a simple and elegant design of a full-subtractor using four single-stranded DNA molecules whereas an electronic full-subtractor in silico would require combinational circuits to perform subtraction. The proposed DNA-based logic operations rely on the interactions among the four DNA strands to switch the gate molecule between hairpin and linear forms and to manipulate the duplex formation of the input strands. These controllable conditions determine the on/off patterns of the fluorescence signals, which correspond to the values in the Boolean truth tables of a full subtractor. The compactness of the proposed design makes it a promising foundation for future applications and integrations with other DNA-based computing devices, although there is a long road ahead toward integrating the developed molecular systems for practical functions and competition with silicon-based technology. In addition to the arithmetic operations, numerous logic gate systems have been synthesized and employed on the DNA sensor development for medicinal applications^17^,^24^. For example, logic gates designed with colorimetric property may have the potential to form intelligent diagnostic devices in response to disease markers. To summarize, the presented work provides a novel prototype for the future circuit design on nanoscale for complicated arithmetic operations. Furthermore, it is also possible to adopt the design concept demonstrated herein along with other arrangement to blueprint new systems for medicinal usage.

## Methods

All of the oligonucleotides (gate molecule, inputs I_A_, I_B_ and B_in_) in the current study were purchased from Yao-Hong Biotechnology Inc. (HPLC grade, New Taipei City, Taiwan). Sodium chloride (A. R. grade), potassium chloride, potassium dihydrogen phosphate (KH_2_PO_4_, >99.9%), and sodium phosphate dibasic (Na_2_HPO_4_, >99%) were purchased from J.T. Baker (Phillipsburg, NJ. USA). Deionized water used in the preparation of phosphate buffered saline (PBS; 110 mM NaCl, 2 mM KCl, 8 mM Na_2_HPO_4_, 2 mM KH_2_PO_4_, pH = 7.4) and for rinse solutions was 18.2 MΩ, and was produced by PURELAB Ultra (ELGA, Albania). All purchased chemicals were used as received, unless noted otherwise.

To prepare the full-subtractor’s gate molecules, 2 μL of 50 μM gate molecules were dissolved in PBS and pipetted on a screen-printed gold substrate with a 4-mm diameter (DropSens, Spain) for immobilization for 30 min. The gate-coated gold surfaces were then rinsed with 50 μL of PBS to remove unbound molecules.

Hybridization of the hairpin probes on the gold surface was performed at room temperature for 30 min, after the same number of molecules (i.e., 2 μL of 50 μM) of (i) I_A_, I_B_, or B_in_, (ii) I_A_+I_B_, I_A_+B_in_ or I_B_+B_in_, (iii) B_in_ added to I_A_+I_B_ molecules was applied under the same buffer conditions. During all processes including gate immobilization and hybridization, all DNA molecules were covered with aluminum foil to prevent photobleaching.

The fluorescence intensities were monitored using a fluorescence spectrophotometer (F7000, Hitachi High-Technologies, Japan), equipped with a solid sample holder. The detector voltage was set at 550 V. The excitation and emission wavelengths were 430, 625 nm, and 520, 662 nm for fluorescein and Cy5, respectively. The peak bandwidths were around ±20~25 nm. The relative intensities of the samples were calculated using I = I_hybrid_–I_blank_, where I_hybrid_ and I_blank_ are the respective fluorescence intensities of the gate molecule on gold after and before hybridization with the target sequences.

**Figure 2:**
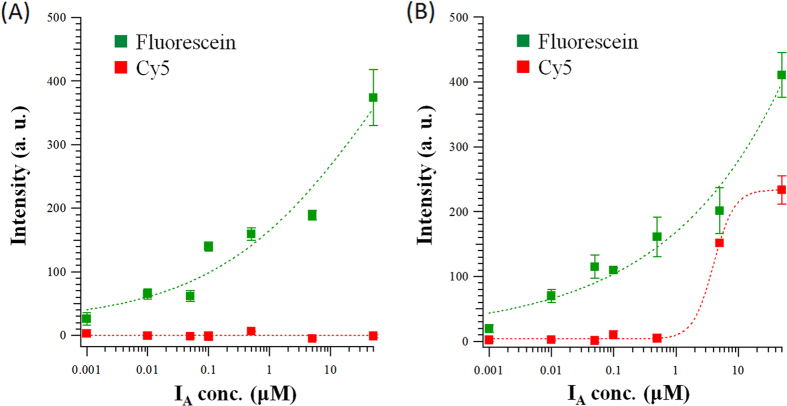
The concentration-dependent sensitivity of I_A_ and I_B_ on the DNA gate coated on the gold surface. (**A**) I_A_’s concentration-dependent sensitivity is between nm and μM. (**B**) Because of low quantum efficiency of Cy5, I_B_’s concentration-dependent sensitivity is around μM.

Figure 2 demonstrates the sensitivity of I_A_ and I_B_ concentrations on the aforementioned gate-coated gold surface. As indicated in Fig. 2(A) where I_A_ strand was added to turn the hairpin shaped gate molecule into linear form to release the fluorescein signal, the concentration-dependent sensitivity is between nm and μM for I_A_. In Fig. 2(B), I_B_ strand was added to transform gate molecule to linear conformation and both the Cy5 and fluorescein signals were observed. Because of low quantum efficiency of Cy5, the concentration-dependent sensitivity of I_B_ is around μM.

**Figure 3:**
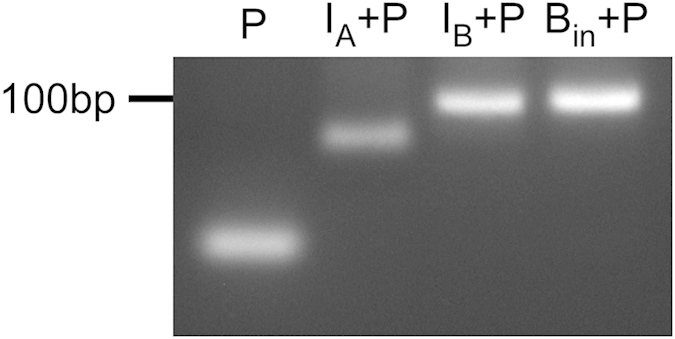
Agarose gel electrophoresis of reaction products. From left to right: lane P, gate molecule (29 nt), lane I_A_ + P, gate molecule reacted with I_A_ (46 nt), lane I_B _+ P, gate molecule reacted with I_B_ (76 nt), and lane B_in_ + P, gate molecule reacted with B_in_ (76 nt). The gate molecule alone and the three combinations underwent in heating and annealing procedure. The reacted products were analyzed by 3% agarose gel electrophoresis and detected by ethidium bromide staining.

A gel electrophoresis experiment was carried out to characterize the hairpin structure of the gate molecule. The mole ratio between gate molecule and I_A_/I_B_/B_in_ is 1:1. After heating and annealing, the reacted product was analyzed by 3% agarose gel electrophoresis. As shown in Fig. 3, the band positions for gate molecule (29 nt) alone in lane P, gate molecule hybridized with I_A_ (46 nt) in lane I_A_+P, gate molecule hybridized with I_B_ (76 nt) in lane I_B_+P, and gate molecule hybridized with B_in_ (76 nt) in lane B_in_+P agree with the mobility due to the size difference. Meanwhile, ethidium bromide staining indicated the gate molecule alone is in hairpin conformation with a stem region to intercalate ethidium bromide.

## Additional Information

**How to cite this article**: Lin, H.-Y. *et al*. A simple three-input DNA-based system works as a full-subtractor. *Sci. Rep*. **5**, 10686; doi: 10.1038/srep10686 (2015).

## Figures and Tables

**Figure 1:**
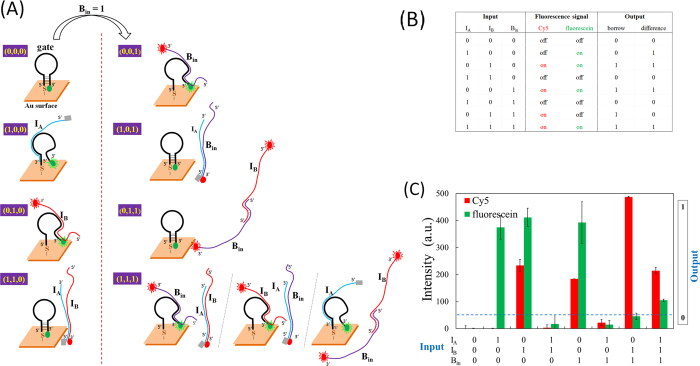
(**A**) A schematic representation of the proposed full-subtractor. The green and red ovals represent the green dye (fluorescein) and the red dye (Cy5), respectively; the gray rectangle represents BHQ3, a quencher to Cy5. (**B**)Truth tables for the eight input states and their matched outputs. (**C**)The observed fluorescence intensity corresponding to the eight input states.

**Table 1:**
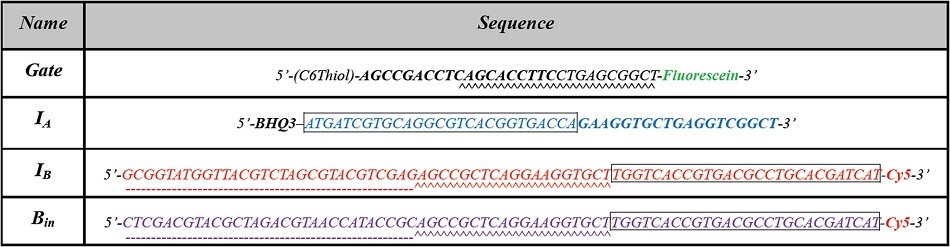
Sequences used in the gate molecule and three inputs including I_A_ (the minuend), I_B_ (the first subtrahend), and B_in_ (the second subtrahend). Colors match the illustrated sequences in Fig. 1(A). Sequences in box, bold font, dashed- or wavy-underlining are complementary to similarly marked sequences. Fluorescein is a green dye; Cy5 is a red dye; BHQ3 is a quencher to Cy5.
